# Excessive erythrocytosis and the hypertensive phenotype at high altitude: emerging evidence and unresolved questions

**DOI:** 10.3389/fcvm.2026.1845046

**Published:** 2026-06-26

**Authors:** Yanan Li, Jun Ma, Xin Zhang, Jialiang Zhang, Xiaoping Chen

**Affiliations:** Department of Cardiology, West China Hospital, Sichuan University, Chengdu, Sichuan, China

**Keywords:** chronic mountain sickness, endothelial dysfunction, excessive erythrocytosis, high-altitude polycythemia, systemic hypertension

## Abstract

Systemic hypertension at high altitude is clinically important but mechanistically heterogeneous. Excessive erythrocytosis (EE), commonly discussed in relation to high-altitude polycythemia (HAPC) or the erythrocytotic component of chronic mountain sickness (CMS), is one of the most visible maladaptive responses to chronic high-altitude hypoxia. Emerging human studies suggest that erythrocytosis burden may be associated with the hypertensive phenotype at high altitude. Here, we critically evaluate the evidence linking EE and systemic hypertension and discuss the principal mechanistic pathways that may connect these phenotypes, including hyperviscosity-related vascular stress, endothelial dysfunction, hypoxemia with sleep-disordered breathing, sympathetic activation, and renal-metabolic disturbance. Current evidence can be organized into three tiers: direct association studies, indirect human physiological studies, and clinical extension studies addressing vascular or organ-damage phenotypes. The evidence base remains limited in sample size, predominantly cross-sectional, and largely restricted to selected high-altitude populations, especially Tibetans. Available data support association more strongly than causation. The key unresolved question is whether EE is a causal determinant of high-altitude hypertension, a marker of more severe hypoxic maladaptation, or a maladaptive amplifier within a broader blood pressure dysregulation phenotype. Clarifying this distinction will require phenotype-rich longitudinal studies integrating hemoglobin burden, blood viscosity, oxygenation, sleep-disordered breathing, ambulatory blood pressure, and vascular phenotyping.

## 1 Introduction

Systemic hypertension at high altitude is increasingly recognized as a clinically relevant phenotype, yet its determinants are more complex than those of hypertension at sea level. In addition to conventional cardiometabolic risk factors, blood pressure regulation at altitude is influenced by chronic hypobaric hypoxia, population-specific adaptive capacity, ventilatory control, endothelial function, and renal-neurohumoral responses (1, 2). Existing reviews therefore frame high-altitude hypertension as a heterogeneous disorder rather than a single-pathway condition (1).

High-altitude exposure is not geographically marginal. Major high-altitude regions include the Tibetan Plateau and Himalayan region in Asia, the Andes in South America, and the Ethiopian and East African highlands in Africa. Depending on the altitude threshold used, approximately 81.6 million people live above 2,500 m, and as many as 842 million people worldwide may live at elevations of 1,500 m or higher (3). In addition to traditional high-altitude populations such as Tibetans, Andean highlanders, and Ethiopian highlanders, the clinical relevance of high-altitude physiology also extends to tourists, migrants, intermittent workers, and other occupationally exposed groups (4).

Although this review focuses primarily on long-term high-altitude residents, non-permanent exposure is also clinically relevant. Short-term sojourners, tourists, soldiers, miners, railway workers, and other occupationally exposed groups differ from lifelong high-altitude residents in the timing and depth of acclimatization (4, 5). During acute ascent, blood pressure regulation is influenced mainly by ventilatory acclimatization, oxygen desaturation, plasma-volume contraction, and sympathetic activation, whereas increased red cell production is not a major early response (4). With repeated or prolonged intermittent exposure, partial acclimatization may occur and may be accompanied by higher hemoglobin or hematocrit and greater cardiopulmonary stress in susceptible individuals (5). Thus, non-permanent and permanent high-altitude populations may share hypoxemia, chemoreflex activation, sleep disturbance, and vascular stress, but the relationship between erythrocytosis burden and the hypertensive phenotype should not be assumed to be identical across long-term high-altitude residents, migrants, and intermittently exposed workers.

Excessive erythrocytosis (EE), commonly discussed as high-altitude polycythemia (HAPC) or as part of chronic mountain sickness (CMS), is one of the most visible maladaptive responses to chronic high-altitude hypoxia. While modest erythrocytosis may support oxygen transport, excessive red cell expansion may lead to hyperviscosity, severe hypoxemia, and broader cardiorespiratory burden. Contemporary work increasingly views EE not as an isolated hematologic abnormality, but as part of a broader maladaptive phenotype involving disordered oxygen homeostasis, vascular dysfunction, and multisystem consequences (6–13).

Despite these parallel lines of research, the relationship between EE and systemic hypertension has not been critically synthesized as a focused question. Earlier reviews addressed high-altitude hypertension without specifically evaluating erythrocytosis burden, whereas HAPC/CMS reviews discussed erythrocytosis primarily in the context of hypoxemia and hyperviscosity (1, 6–8). More recent human studies, however, have reported direct positive associations between HAPC, higher hemoglobin burden, and hypertension risk in Tibetan populations (14, 15). These observations suggest that erythrocytosis may be relevant to the hypertensive phenotype at altitude, but they do not yet establish whether EE is a cause, a marker of more severe maladaptation, or a maladaptive amplifier within a broader blood pressure dysregulation phenotype.

This Mini Review critically evaluates the evidence linking EE and systemic hypertension at high altitude, highlights the mechanistic links most strongly supported by human data, and outlines the unresolved questions most relevant to hypertension-focused research.

## 2 Scope and conceptual framework

In this Mini Review, we focus on erythrocytosis burden at high altitude and its potential relationship with the hypertensive phenotype. For simplicity, we use excessive erythrocytosis (EE) as an umbrella term for maladaptive elevations in hemoglobin concentration or red cell mass during chronic high-altitude exposure. In Chinese clinical studies, HAPC is commonly operationalized using sex-specific hemoglobin thresholds of ≥210 g/L in men and ≥190 g/L in women among long-term high-altitude residents (7). In the international high-altitude literature, CMS refers more broadly to a chronic maladaptation syndrome in residents above 2,500 m, typically characterized by excessive erythrocytosis together with hypoxemia-related symptoms and signs (6, 8). Here, our interest is not the full CMS syndrome, but specifically the extent of erythrocytosis itself as a clinically visible maladaptive phenotype.

This distinction is important because erythrocytosis at high altitude does not appear to behave as a simple binary condition. Rather, available studies suggest a continuum ranging from adaptive hemoglobin elevation to excessive, maladaptive erythrocytosis associated with hyperviscosity, hypoxemia, and vascular dysfunction (6, 7, 9). For conceptual clarity, we focus on erythrocytosis attributable to high-altitude exposure rather than secondary erythrocytosis caused by chronic lung disease, renal disease, hematologic disorders, or other non-altitude conditions.

This review is restricted to systemic hypertension rather than pulmonary hypertension. Our working framework is that chronic high-altitude hypoxia may promote a cluster of maladaptive responses—including excessive erythrocytosis, impaired vascular function, hypoxemia, and neurohumoral disturbance—and that erythrocytosis burden may function as a marker, modifier, or amplifier of a broader hypertensive phenotype rather than as a uniformly causal factor.

## 3 Why this question is biologically and clinically plausible

A plausible EE-hypertension link begins with hemorheology. In maladaptive high-altitude erythrocytosis, hemoglobin concentration, hematocrit, and blood viscosity all increase, potentially impairing microcirculatory flow and increasing vascular load (7, 16, 17). From a blood pressure perspective, this provides a reasonable basis for increased peripheral resistance and reduced hemodynamic reserve. Hyperviscosity is therefore not simply a hematologic epiphenomenon; it may be one mechanism by which erythrocytosis burden becomes hemodynamically relevant (6, 17).

A second basis for plausibility comes from vascular phenotype studies. CMS and EE have been associated with systemic vascular dysfunction, including impaired endothelial-dependent vasodilation and broader vascular abnormalities (16, 18). These observations matter because endothelial dysfunction and loss of vasodilatory reserve are highly relevant to abnormal vascular tone and blood pressure regulation. Human physiological work further shows that high-altitude residents with EE have impaired sustained-stimulus flow-mediated dilation, and that higher hemoglobin concentration and blood viscosity contribute to this abnormal endothelial response (18). Together, these findings suggest that erythrocytosis burden may be linked to blood pressure abnormalities not only through passive rheological stress, but also through active disruption of vascular adaptation.

A third reason this question warrants attention is that EE often coexists with disturbances already implicated in the hypertensive phenotype at altitude, especially chronic hypoxemia, sleep-disordered breathing, and sympathetic activation (1, 7, 19). CMS/high-altitude studies have shown that sleep-disordered breathing is associated with vascular dysfunction, supporting the view that EE may be part of a broader phenotype of disordered oxygenation and vascular stress rather than an isolated hematologic abnormality (19).

Accordingly, the relevance of EE to hypertension research does not depend on proving a one-step causal pathway. Even if erythrocytosis is not an independent cause of hypertension, it may still identify a clinically important maladaptive phenotype characterized by greater vascular stress and more adverse blood pressure regulation.

## 4 Current human evidence: what is established, and what remains uncertain?

Current evidence linking EE and the hypertensive phenotype at high altitude can be organized into three tiers: Tier 1, direct association studies; Tier 2, indirect human physiological studies; and Tier 3, clinical extension studies addressing vascular or organ-damage phenotypes. This structure is useful because the literature remains limited in sample size, predominantly cross-sectional, and highly population-specific (Table 1).

**Table 1:** Direct and indirect human evidence linking excessive erythrocytosis, hemoglobin burden, and the hypertensive phenotype at high altitude.

Evidence tier	Study	Population/altitude	Design	Exposure	Outcome	Main finding	Key limitation
Tier 1: Direct association	Yin et al. (14)	387 adults; Tibet, 4,700 m	Cross-sectional	HAPC; hemoglobin continuous	Hypertension	HAPC was associated with hypertension; each 10 g/L increase in Hb was associated with higher hypertension risk.	Cross-sectional; single Tibetan ultrahigh-altitude cohort
Tier 1: Direct association	Liu et al. (15)	Native Tibetans; ∼3,500 m	Cross-sectional	Hemoglobin, mild polycythemia, HAPC	Hypertension risk	Risk increased across Hb burden; threshold analysis suggested an inflection point near 176 g/L; milder erythrocytosis and overt HAPC were associated with higher hypertension risk.	Cross-sectional; limited generalizability beyond Tibetans
Tier 2: Indirect human physiology	Rimoldi et al. (16)	CMS patients and controls	Comparative physiology	CMS/EE phenotype	Systemic vascular function	CMS linked to endothelial and systemic vascular dysfunction.	No incident-hypertension endpoint
Tier 2: Indirect human physiology	Tremblay et al. (18)	Andean highlanders with and without EE; 4,330 m	Comparative physiology	EE; blood viscosity; hemoglobin	Sustained-stimulus FMD	Higher Hb and viscosity impaired endothelial-dependent vasodilation.	Small physiology study; no BP outcome
Tier 2: Indirect human physiology	Rexhaj et al. (19)	CMS and healthy high-altitude dwellers	Comparative physiology	Sleep-disordered breathing in CMS/high altitude	Vascular function	Nocturnal hypoxemia/SDB linked to vascular dysfunction.	Indirect relevance to systemic hypertension
Tier 3: Clinical extension	Yang et al. (20)	88 hypertensive high-altitude residents	Matched case-control	HAPC; hemoglobin	Hypertension-mediated organ damage	Hb linked to impaired FMD and larger LA diameter.	No incident-hypertension analysis

CMS, chronic mountain sickness; EE, excessive erythrocytosis; FMD, flow-mediated dilation; HAPC, high-altitude polycythemia.

### 4.1 Tier 1: Direct association studies

The most direct evidence comes from recent studies in Tibetan high-altitude populations. In ultrahigh-altitude residents of Anduo County, Tibet (4,700 m), Yin et al. reported that HAPC was positively associated with hypertension, and that hemoglobin analyzed continuously was also associated with higher odds of hypertension (14). This study currently represents the clearest direct human evidence that erythrocytosis burden may be linked to systemic hypertension at ultrahigh altitude.

A complementary study in native Tibetans found that higher hemoglobin concentration was associated with greater hypertension risk, with evidence of a graded relationship and a possible threshold effect near 176 g/L (15). Importantly, the signal was not limited to overt HAPC; risk appeared to increase along a continuum of rising hemoglobin burden. These findings broaden the question from dichotomous disease classification to exposure-response interpretation.

However, these direct association studies should be interpreted cautiously. Both are cross-sectional, and both are largely confined to Tibetan populations at very high altitude (14, 15). As a result, they cannot establish temporal sequence, cannot exclude reverse causation, and may be vulnerable to residual confounding. Moreover, we did not identify large longitudinal cohort studies specifically designed to test whether erythrocytosis burden predicts incident systemic hypertension at high altitude. Thus, current human evidence supports association more strongly than causation.

### 4.2 Tier 2: Indirect human physiological studies

Indirect human evidence strengthens the biological credibility of this association. Physiological studies show that high-altitude residents with EE have impaired endothelial-dependent vasodilatory responses, and that higher hemoglobin concentration and blood viscosity contribute to this abnormality (18). Earlier work in CMS likewise demonstrated systemic vascular dysfunction rather than a phenotype confined to pulmonary or hematologic abnormalities (16). Rexhaj et al. further showed that sleep-disordered breathing in CMS/high-altitude populations is associated with vascular dysfunction (19). Collectively, Tier 2 evidence suggests that erythrocytosis burden is not merely an isolated hematologic marker, but may identify a physiologically unfavorable phenotype in which vascular and oxygenation-related stressors converge.

### 4.3 Tier 3: Clinical extension studies

Additional extension evidence comes from studies of hypertensive high-altitude patients showing that HAPC may be associated with worse endothelial function and less favorable cardiovascular structural phenotypes (20). These observations do not prove that EE increases the incidence of hypertension, but they do suggest clinical relevance within established hypertensive populations.

### 4.4 Discordant and non-uniform observations

Preliminary data from some Tibetan high-altitude communities have shown that polycythemia and hypertension do not necessarily rise in parallel (21). More broadly, altitude-related blood pressure patterns differ across Tibetan and non-Tibetan high-altitude populations (22). This non-uniformity is biologically plausible. Tibetans, Andean highlanders, and Ethiopian highlanders differ in hemoglobin distribution, ventilatory adaptation, nitric oxide (NO) biology, and vascular physiology (23–32). Therefore, the blood pressure significance of a given erythrocytosis burden is unlikely to be identical across all high-altitude populations.

Taken together, current human evidence—dominated by cross-sectional Tibetan studies and supported by indirect human physiological data—supports association more strongly than causation. The literature currently lacks large, phenotype-rich longitudinal studies integrating hemoglobin burden, blood viscosity, oxygenation, sleep-disordered breathing, ambulatory blood pressure, endothelial function, and renal-metabolic markers. Until such studies are available, the EE-hypertension link should be regarded as plausible and clinically relevant, but not yet definitive.

## 5 Mechanistic links: which pathways are best supported?

**Figure 1:**
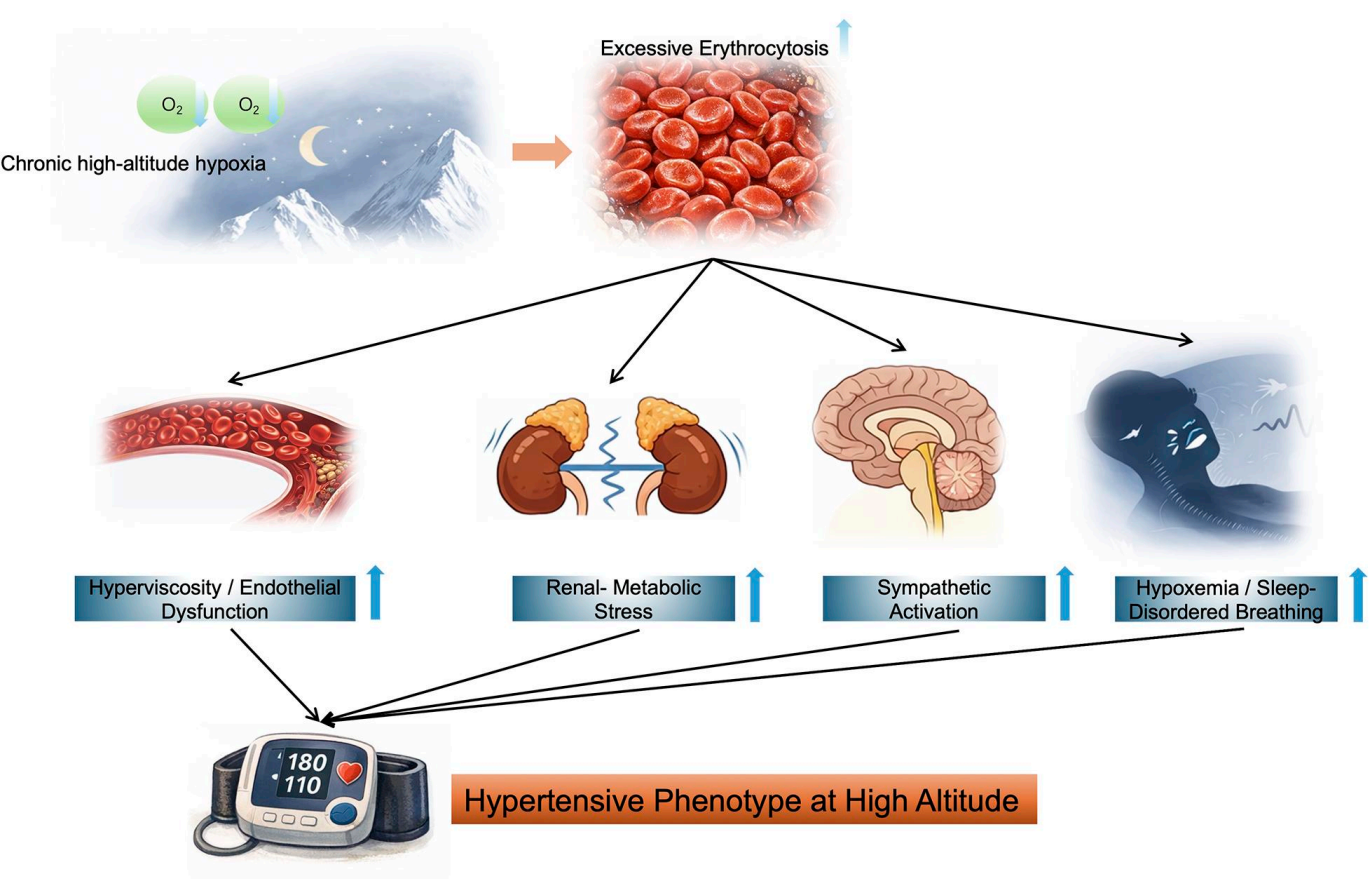
Proposed mechanistic framework linking excessive erythrocytosis to the hypertensive phenotype at high altitude. Chronic high-altitude hypoxia may increase erythrocytosis burden and promote hyperviscosity, endothelial dysfunction, hypoxemia with sleep-disordered breathing, sympathetic activation, and renal-metabolic stress, thereby increasing susceptibility to an adverse hypertensive phenotype.

Mechanistic interpretation of the EE-hypertension link is best approached by separating pathways supported by direct human evidence from those supported mainly by indirect or translational evidence. The proposed mechanistic framework is summarized in Figure 1.

### 5.1 Pathways supported by direct human evidence

Hyperviscosity-related vascular stress is the most direct mechanistic candidate. Excessive erythrocytosis increases hemoglobin concentration and hematocrit, thereby increasing blood viscosity and potentially raising peripheral vascular load (6, 17, 18). This may impair microcirculatory flow, reduce perfusion reserve, and increase hemodynamic stress across the systemic circulation. Hyperviscosity is therefore the most intuitive bridge between erythrocytosis burden and blood pressure elevation. Yet hyperviscosity alone is unlikely to explain the full phenotype, because a more uniform EE-hypertension relationship would otherwise be expected across populations (1, 20).

Endothelial dysfunction and impaired vasodilatory reserve constitute a second major pathway. Human studies in EE and CMS show impaired endothelial-dependent vasodilatory responses and broader systemic vascular dysfunction (16, 18). These abnormalities are directly relevant to blood pressure regulation because reduced endothelial reserve may favor higher resting vascular tone and impaired accommodation to hypoxic and hemodynamic stress. Among currently proposed mechanisms, hyperviscosity-related vascular stress and endothelial dysfunction are the best supported by human data.

### 5.2 Pathways supported by indirect human evidence

EE often coexists with marked hypoxemia and ventilatory instability; in turn, nocturnal hypoxemia and breathing instability may promote sympathetic activation, vasoconstrictor tone, and abnormal nocturnal blood pressure regulation (1, 7, 19). Hypobaric hypoxia stimulates peripheral chemoreceptors, particularly the carotid bodies, leading to increased sympathetic outflow, higher heart rate, enhanced vasoconstrictor tone, and greater blood pressure variability (1, 4, 5). EE may interact with this pathway indirectly: higher hemoglobin and hematocrit increase blood viscosity and vascular load, whereas more severe hypoxemia may strengthen chemoreflex drive (6, 17, 18). The coexistence of hyperviscosity, impaired endothelial vasodilatory reserve, and sympathetic vasoconstriction may therefore favor higher systemic vascular resistance. However, direct human studies simultaneously assessing sympathetic activity, erythrocytosis burden, oxygenation, and ambulatory blood pressure remain scarce.

High-altitude blood pressure regulation is also influenced by endothelial NO signaling. Endothelial NO synthase (eNOS) generates NO from L-arginine, and NO promotes vasodilation through the soluble guanylyl cyclase–cyclic guanosine monophosphate pathway in vascular smooth muscle (1, 33–35). Hypoxia may reduce NO bioavailability through altered eNOS activity, oxidative NO inactivation, impaired substrate or cofactor availability, asymmetric dimethylarginine-related NOS inhibition, altered shear stress, and hemoglobin-mediated NO scavenging (33–35). Because high-altitude populations differ in NO biology, this pathway may help explain why the blood pressure significance of a given hemoglobin or hematocrit level differs across populations. Nevertheless, direct human studies linking NO-related biomarkers, erythrocytosis burden, and systemic blood pressure remain limited.

### 5.3 Pathways supported mainly by translational or syndrome-level evidence

A further mechanistic interface may involve renal and metabolic pathways. High-altitude maladaptation syndromes have been described in which polycythemia, hypertension, albuminuria, and hyperuricemia cluster together, suggesting that renal perfusion abnormalities, altered sodium-volume handling, or metabolic stress may participate in the EE-hypertension relationship (36, 37). Compared with hyperviscosity or endothelial dysfunction, this pathway remains less directly supported in human mechanistic studies.

A limited degree of discussion from basic and translational research also helps contextualize the EE-hypertension link. Chronic hypoxia is likely to activate overlapping pathways that influence both erythrocytosis and blood pressure regulation, including NO dysregulation, altered vasoconstrictor signaling, hypoxemia-related sympathetic activation, and renal-metabolic stress (1, 7, 13). At present, these pathways are better supported as biologically plausible mechanisms than as directly validated causal links in human EE-associated hypertension.

These pathways are unlikely to operate independently. Hyperviscosity may aggravate endothelial dysfunction, while hypoxemia and sleep-disordered breathing may amplify both neurohumoral stress and the hemodynamic consequences of erythrocytosis burden. Thus, current mechanistic evidence is best viewed as a network of interacting processes rather than a single dominant pathway.

## 6 Discussion

### 6.1 Unresolved questions

One of the main reasons the EE-hypertension relationship remains unsettled is heterogeneity. High-altitude populations differ substantially in genetic background, ventilatory adaptation, NO biology, hemoglobin distribution, and susceptibility to maladaptive erythrocytosis (1, 6, 23–32). Contextual modifiers such as age, obesity, smoking, renal dysfunction, altitude level, and conventional cardiometabolic risk factors are also likely to matter.

The major unresolved question is whether EE is a causal determinant, a marker of more severe hypoxic maladaptation, or a maladaptive amplifier of the hypertensive phenotype at altitude. A second issue concerns the most informative exposure metric. Recent human data suggest that hypertension risk may increase across the continuum of hemoglobin burden rather than only after classic HAPC thresholds are reached (14, 15). In native Tibetans, one study identified a possible inflection point near 176 g/L, above which the hemoglobin-hypertension association became more pronounced (15). In adults at Tibetan ultrahigh altitude, HAPC and higher hemoglobin analyzed continuously were also associated with greater hypertension risk (14). However, hemoglobin alone is unlikely to capture the full hemodynamic effect of erythrocytosis, because hematocrit, plasma volume, blood viscosity, oxygenation, sleep-disordered breathing, endothelial function, and renal-metabolic status may all modify risk (17–19, 36, 37). Future studies should therefore compare hemoglobin, hematocrit, measured viscosity, and composite maladaptation indices for predicting ambulatory blood pressure, incident hypertension, and hypertension-mediated organ damage.

### 6.2 Future directions

Addressing the unresolved role of EE will require prospective cohort studies with repeated blood pressure phenotyping and incident hypertension as an outcome. Interventional studies examining whether reducing erythrocytosis burden or improving oxygenation alters blood pressure trajectories would be even more informative.

In addition, phenotyping needs to move beyond office blood pressure alone. Future studies should integrate hemoglobin burden, direct viscosity measurements, oxygen saturation, ambulatory blood pressure monitoring, sleep assessment, endothelial or vascular phenotyping, and renal-metabolic markers within the same cohorts. Because high-altitude hypertension may involve altered nocturnal physiology, oxygenation instability, and early vascular injury, ambulatory blood pressure monitoring, nighttime blood pressure patterns, and hypertension-mediated organ damage should be incorporated whenever feasible (1, 19, 20).

Replication in Andean highlanders, Ethiopian highlanders, long-term migrants, and other high-altitude groups is essential to determine whether the EE-hypertension relationship is biologically uniform or adaptation-specific. Population-specific adaptation deserves special emphasis because Tibetan and Andean highlanders differ in hemoglobin distribution, ventilatory phenotype, NO biology, and likely in the hemodynamic consequences of erythrocytosis (23–26, 32). This heterogeneity argues against adopting a single universal interpretation of erythrocytosis burden in hypertension research.

### 6.3 Clinical implications

Even without definitive causal proof, the available evidence has practical implications. Erythrocytosis burden may help identify high-altitude residents who are more likely to exhibit adverse blood pressure phenotypes or concurrent vascular dysfunction. At present, the most defensible clinical message is that EE should be regarded as a potentially relevant component of high-altitude cardiovascular maladaptation and a phenotype worthy of attention in blood pressure assessment and future intervention-oriented research. In clinical settings, marked erythrocytosis may justify closer blood pressure monitoring and greater attention to oxygenation status, sleep-disordered breathing, endothelial dysfunction, and blood viscosity, although these suggestions still require validation in prospective studies.

## 7 Conclusion

Available human evidence suggests that excessive erythrocytosis and higher hemoglobin burden are associated with the hypertensive phenotype in selected high-altitude populations, particularly Tibetan cohorts, and that this association is biologically reinforced by concurrent vascular dysfunction observed in EE and CMS phenotypes. Among the proposed mechanisms, hyperviscosity-related vascular stress and impaired endothelial vasodilatory reserve currently have the strongest support from human data, whereas hypoxemia-related instability, neurohumoral stress, NO-related pathways, and renal-metabolic interfaces remain important but less directly substantiated.

At the same time, the field remains far from settled. The current literature is dominated by cross-sectional studies, meaningful population heterogeneity exists, and the distinction between causation, association, and maladaptive clustering has not been resolved. A defensible interpretation at present is that erythrocytosis burden identifies a clinically relevant maladaptive phenotype that may amplify blood pressure dysregulation in selected high-altitude populations, but is not yet a definitively proven cause of systemic hypertension.

Future progress will depend on moving beyond isolated hemoglobin measurements toward integrated phenotyping strategies that combine blood pressure profiling, oxygenation status, ventilatory function, vascular assessment, and renal-metabolic characterization across multiple high-altitude populations. Such work will be necessary to determine whether excessive erythrocytosis is simply a visible sign of maladaptation or a modifiable contributor to high-altitude hypertension.
