# Correction: Excessive erythrocytosis and the hypertensive phenotype at high altitude: emerging evidence and unresolved questions

**DOI:** 10.3389/fcvm.2026.1923690

**Published:** 2026-07-14

**Authors:** Yanan Li, Jun Ma, Xin Zhang, Jialiang Zhang, Xiaoping Chen

**Affiliations:** Sichuan University West China Hospital Department of Cardiology, Chengdu, China

**Keywords:** chronic mountain sickness, endothelial dysfunction, excessive erythrocytosis, high-altitude polycythemia, systemic hypertension

Jialiang Zhang was erroneously omitted as corresponding author. The correct corresponding authors are Jialiang Zhang and Xiaoping Chen.

The original version of this article has been updated.

